# Nurses and Managers’ Time Management Skills Assessment: A National Survey in the Italian Healthcare Setting

**DOI:** 10.3390/nursrep14030157

**Published:** 2024-08-23

**Authors:** Lucia Filomeno, Yassin Chaoui, Antonietta Scinicariello, Andrea Minciullo, Sofia Di Mario

**Affiliations:** 1 Department of Biomedicine and Prevetion, University Tor Vergata, 00133 Rome, Italy; lucia.filomeno@uniroma1.it; 2 Department of Public Health and Infectious Diseases, Sapienza University of Rome, 00185 Rome, Italy; chaoui.yassin96@gmail.com; 3 Azienda Ospedaliera San Giovanni Addolorata, 00184 Rome, Italy; antoscinicariello@libero.it; 4 Fondazione Policlinico Campus Bio-Medico University Hospital, 00128 Rome, Italy; a.minciullo@policlinicocampus.it

**Keywords:** nurses, attitudes, knowledge, time management, skills

## Abstract

One of the key strategies for the success of health organizations currently resides in the ability to develop advanced competencies in time management. Individuals who are able to spend their time efficiently are those who do not focus on a single issue within the allotted time but rather spread their time among several tasks. This study aims to investigate the attitudes, beliefs and knowledge towards the time management of nurses (clinicians, first-line and middle-level nurse managers) in their daily work. A descriptive, cross-sectional survey was conducted in private and public settings across Italy. Time management was assessed using the University “G. D’Annunzio” of Chieti—Laboratory of Business Psychology’s Questionnaire. Among the respondents (N = 74), 67.6% were female, and the age range was 51–60 years (40.5%). The three reported sections (Time management, Health conditions and Ability to delegate) showed several items with statistical significance (*p* < 0.05). Anxiety, stress and negative perceptions are statistically related to time management skills and knowledge. Healthcare institutions and regulatory bodies should provide resources and support to nurses and managers to improve their time management. The topic is of paramount importance and forms the basis of all work performed.

## 1. Introduction

The continuous and daily management of numerous information and data, managing and meeting deadlines, checking and organizing services and activities and being efficient and productive are all tasks that a nurse must take charge of, and that involves an expenditure of time [1]. Management studies have the common purpose of helping those who lead companies to meet human needs and work efficiency in a scenario characterized by unexpected changes in economic and social conditions [2].

The concept of time management (TM) started with the industrial revolution and has now become the modern notion of doing things effectively and efficiently [3]. It is difficult to measure the practice of time management, but this largely depends on the performance results of the workers [4]. Good TM means doing high-quality work, not a large quantity.

The scientific community’s first studies and interest towards TM began to develop in the 1960s [1]. Peter F. Drucker in 1967 [4] and Alan Lakein in 1973 [5] were considered the most influential management thinkers ever. Through their papers, they identified and proposed to the professional executive community, methods and remedies to increase work performance such as scheduling and filling in lists or checklists for the systematic and regular management of tasks and activities. The author who most of all played a central role in the development of TM techniques was James T. McCay, who in 1959 [6] first developed and implemented various programs of “management-training” aimed at increasing the efficiency of work performance by using as a fundamental tool: the scheduling and structuring of daily plans of activities to be carried out, assigning priority degrees to different activities and defining in advance the methods of managing contingencies [7].

Over the years, many scholars have defined the construct of time management in different forms: “a process in which the needs are determined by assigning priorities and planning the tasks necessary to meet them” [6], “the degree to which individuals perceive their use of time to be systematic and purpose-oriented” [8], “time management techniques” [9,10], and a “set of time-oriented behaviours when performing activities for a purpose” [1].

The first scientific articles on time management were mainly of an informative nature. A preliminary review of the literature provided a framework on the current state of the art, highlighting how there is still a small number of scientific articles related to the tools for measuring the management of TM and related to theoretical and practical models of reference that explain the operation and applicability of such techniques [11].

Time management in nursing is a critical skill that significantly impacts the quality of patient care, the efficiency of healthcare delivery, and the well-being of nurses themselves. In the dynamic and often high-pressure environment of healthcare, nurses are required to juggle multiple tasks, make quick decisions, and prioritize patient needs—all while maintaining a high standard of care. Effective time management in nursing not only enhances patient outcomes but also helps in reducing the stress and burnout that are prevalent in the profession [8,9,10,11].

Nursing is a profession characterized by its demanding nature and the need for meticulous attention to detail. Nurses are responsible for administering medications, monitoring patient vitals, updating medical records, coordinating with other healthcare professionals, and providing emotional support to patients and their families. Given the breadth of these responsibilities, efficient time management is essential [2,3].

Proper time management allows nurses to allocate their time and resources effectively, ensuring that they can meet the needs of all their patients without compromising the quality of care. It helps in prioritizing critical tasks, thereby reducing the likelihood of errors, which can have serious implications for patient safety. Additionally, good time management can lead to improved job satisfaction, as nurses are able to complete their duties more efficiently and with less stress [10].

**Strategies for Effective Time Management in Nursing**

Several strategies can enhance time management skills in nursing. One effective method is the use of clinical pathways and care plans, which provide a standardized approach to patient care. These tools help in streamlining processes and ensuring that all necessary steps are taken in a timely manner [10].

Another strategy is the implementation of time audits. By regularly reviewing how time is spent during shifts, nurses can identify areas where they can improve efficiency and reduce time wastage. This reflective practice can lead to better time allocation and task management [10].

Effective communication is also paramount. Clear and concise communication with colleagues, patients, and families can prevent misunderstandings and delays, thereby saving valuable time. Utilizing handoff reports and interdisciplinary team meetings can ensure that everyone is on the same page and tasks are clearly defined and understood [10].

Additionally, self-care and stress management are crucial for maintaining the stamina needed for effective time management. Nurses who are well rested and manage their stress levels are better equipped to handle the demands of their job efficiently [10,12].

To our knowledge, a gap persists in achieving desired productivity and improving the performance of middle managers [11].

The aim of this study is to investigate attitudes, knowledge and skills towards time management among nurses and nurse managers.

## 2. Methods

### 2.1. Study Design

This study is a descriptive, cross-sectional, nationwide survey conducted to investigate nurse managers’ and nurses’ knowledge and attitudes on time management. Strengthening the Reporting of Observational studies in Epidemiology (STROBE) guidelines were followed throughout [13]. A STROBE checklist was completed and included as a Supplementary File. The questionnaire was administered through an online platform to healthcare professionals. The questionnaire took approximately 10 min to complete. The links expired after the completion of the survey, thus not allowing multiple answers from the same user. All questions were set as mandatory fields with automated skip logic to prevent missing data and avoid illogical or incompatible answers.

### 2.2. Participants

All healthcare professionals other than nurses were excluded from the final sample.

A convenience sample of RNs, Ans and MNMs was recruited. Nurses could participate in this study if they met the following inclusion criteria: (1) worked as clinical nurses on a regular basis, and (2) had at least 1 year of working experience. Researchers identified and then met with potential participants to explain the aims of the research and the data collection procedure prior to the administration of the instruments. No setting restrictions were applied.

### 2.3. Variables and Measurement

The online survey consisted of six main sections: sociodemographic information, occupation and work history, the 39-item version of the Time Structure Questionnaire—TSQ [8], the 14-item Time Management Questionnaire TMQ [14], the 7-item Job Content Questionnaire—JCQ of Karasek [15], the 32-item WART—Work Addiction Risk [16] and the 5-item WFC—“Work-Family Conflict” [17]. Common features of the three tools are the multidimensionality of the time management construct and the inclusion of items related to planning behaviors: “Setting goals and priorities”, “Systematic routine”, “Short- and long-term planning”. These questionnaires were used in an aggregate form that makes up a single tool validated in Italian by the “G. D’Annunzio” University of Chieti—Laboratory of Business Psychology. The final questionnaire assessed dimensions such as job insecurity, coworker support, social support, job demand, decision latitude, speed and impatience, job involvement, competition, emotional charge, supportive management and supportive colleagues, setting goals and priorities, planning and scheduling, perceived control of time and time flexibility.

### 2.4. Ethical Considerations

Before filling in the questionnaire, all participants had to read a brief description of the research and information on the privacy and anonymity of data processing per EU Regulation 2016/679.

Ethics approval was not applicable to this study as it does not involve any personally identifiable information. This study was conducted according to the principles of the Declaration of Helsinki and in accordance with the Checklist for Reporting Results of Internet E-Surveys (CHERRIES Statement) [18]. Respondents were free to refuse to participate or withdraw from this study at any time.

### 2.5. Questionnaire

The questionnaire consists of two sections: sociodemographic information, which investigates the main characteristics of the survey sample (age, sex, marital status, children, education, region, etc.), and a second section comprising knowledge, attitudes and behaviors, skills, appropriate use of time management actions and tools during the professional and personal routine which is investigated through 69 items with a 5-points Likert scale (agreement/disagreement to the statements exposed). The University “G. D’Annunzio” of Chieti—Laboratory of Business Psychology’s time and work management questionnaire was administered.

### 2.6. Data Collection

This research study involved 74 respondents and was conducted by administering an anonymous questionnaire between October 2022 and January 2023. Data were gathered via an online survey hosted on the Google Form platform. Responders were approached via email that featured a link to the online survey, which collected demographic information and included questions. Also, social media outlets (Twitter, Instagram and LinkedIn) were used to share the link to the survey.

### 2.7. Statistical Analysis

The data were analyzed using descriptive and inferential statistics with IBM SPSS Statistics software (version 26.0) (IBM, Armonk, NY, USA). Means, standard deviations, frequencies, and percentages for sociodemographic factors such as gender, age, and employment status were determined. In addition, percentages were generated to compare differences across subgroups (for example, male/female, younger/older, and ward/outpatient).

The survey responses were analyzed using Pearson’s χ2 statistics, a test for categorical data, to identify differences depending on gender, age, and work environment. The χ2 test is the most used, providing thorough information on the categories responsible for potential variances. The minimum level of significance accepted was *p* ≤ 0.05.

## 3. Results

A total of 74 questionnaires were collected. No missing data were recorded. The following characteristics of the sample were taken into account: gender, age, profession, education, type of work, and whether they were parents or not. The sample of participants who completed the survey was predominantly female (67.6%). According to the role covered, 75.7% of the sample were registered nurses, 12.2% were middle nurse managers, and 8.1% were nurse directors. Most of the sample (97.3%) worked full-time, and 78.4% worked in a public hospital. The age reported was predominantly in the 51–60 category (40.5%), followed by the 31–40 group (21.6%), the 41–50 group (18.9%), the <30 group (10.8%) and concluding with the 61–70 category (8.1%). The sociodemographic characteristics of the participants are presented in Table 1.

After analyzing the demographic variables of the sample, the remaining items can be categorized into three different sections: a first section concerning time management, a second concerning the health condition of the participants in the last two weeks, and finally, a third section analyzing the ability to delegate. Additional analyses were conducted using correlations. Pearson’s Chi-Square test was used for the dichotomous variables. The 0.05 significance level was used for this study with a two-tailed significance test. Pearson’s correlations showed that there was statistical significance.

### 3.1. Time Management

Table 2 summarized the first section. From the main results of the first section of the questionnaire, it can be seen that most participants completely agreed to break down more complex projects into smaller tasks (60.8%). The participants agreed (68.9%) to impose deadlines on themselves before starting a task and to impose priorities with which the different tasks will be carried out (75.6%). Moreover, participants are indifferent about planning tasks a week in advance (37.9%) and about stating that they are in control of their time (40.5%). Participants disagree in stating that workdays are so unpredictable that they cannot plan work (43.2%) and spend a lot of time on unimportant tasks (55.4%).

For this section, there is much statistical significance between job position and the question “*I ask myself when I set out to accomplish a task*” (*p* value of 0.006); work position and the question “*my work days are so unpredictable that I am unable to plan and manage my time in the long term*” (*p* value of 0.005); gender and the question “*I set my priority to determine the time order in which I will carry out different tasks*” (*p* value of 0.01); part-time or full-time work and the question “*my days are so unpredictable that I cannot plan and manage my time in the long term*” (*p* value of 0.003); and educational qualification and the question “*I have to spend a lot of my time on unimportant tasks*” (*p* value of 0.05).

### 3.2. Participants Well-Being

The second part of the questionnaire concerns the health condition of the participants in the last two weeks. Table 3 summarized the main results. The sample analyzed expressed that they had problems with concentration and loss of sleep as usual, that they had no feeling at all that they were performing activities that were not useful for their role or that they had difficulty in making decisions. The sample reported feeling under stress and having difficulty overcoming problems. Most of the participants stated that they were neither unhappy nor depressed, and half of the sample stated that they had lost their self-confidence on some occasions.

For this section, there are also many statistical significances between job position and the feeling of performing unhelpful activities (*p* value of 0.004), and the lack of pleasure in performing many activities (*p* value of 0.01). Between gender and the lack of pleasure in performing many activities (*p* value of 0.02). Between having children and the feeling of doing unhelpful activities (*p* value of 0.01) and doing unhelpful activities (*p* value of 0.01), and the difficulty in overcoming problems (*p* value of 0.05).

### 3.3. Ability to Delegate

The third part concerned the ability to delegate (Table 4). Most nurses never delegate. When the task is related to their priorities, most avoid delegating it to third parties, and some of them use delegation as a means of developing the competencies and skills of their employees.

For the last section, there is one significative correlation, between educational qualification and the use of delegation as a means of developing employees’ skills (0.002).

## 4. Discussion

The present study assessed attitudes, knowledge and skills towards time management in 74 nurses. This survey showed that the topic of time management for middle managers is important to evaluate.

Effective time management is an essential skill for nurses, directly influencing the quality of patient care, the efficiency of healthcare services, and the personal well-being of the nurses themselves. In this discussion, we explore the various aspects of time management in nursing, including its challenges, strategies and impacts, supported by relevant literature.

**Challenges in Time Management**

Nurses face numerous challenges in managing their time effectively. The unpredictable nature of patient care, frequent interruptions, and the high volume of administrative tasks are significant obstacles. According to a study by Häggström et al. (2012), interruptions and unforeseen events are common in nursing, leading to increased stress and decreased efficiency. These disruptions often result in tasks being left incomplete or rushed, compromising the quality of care and increasing the potential for errors [19].

**Strategies for Effective Time Management**

To address these challenges, nurses can employ several strategies. Prioritization of tasks is crucial, allowing nurses to focus on the most critical and time-sensitive activities. The use of tools such as the Eisenhower Matrix can aid in distinguishing between urgent and important tasks [20]. Additionally, the implementation of clinical pathways and standardized care plans can streamline workflows and reduce the time spent on decision-making.

Like the results of our study demonstrated, delegation is another key strategy. Effective delegation involves assigning tasks to team members based on their skills and competencies, ensuring that the workload is distributed appropriately [21]. This not only helps manage time more efficiently but also enhances team collaboration and the overall quality of care.

**Impact on Patient Care and Nurse Well-Being**

Effective time management positively impacts both patient care and nurse well-being. By managing their time efficiently, nurses can ensure that they provide comprehensive and attentive care to each patient. This not only improves patient outcomes but also enhances patient satisfaction [22,23].

Furthermore, good time management reduces stress and burnout among nurses. Burnout is a prevalent issue in nursing, often resulting from excessive workload and time pressures [24]. Our survey proved that by employing effective time management strategies, nurses can achieve a better work–life balance, leading to increased job satisfaction and reduced turnover rates.

## 5. Limitations

Despite the fact that the phenomenon of time management has been increasingly studied in recent years, it still remains a subject that is little analyzed in all its facets. The limited number of participants who participated in this study may not make the results generalizable to the entire population, so a larger sample size is recommended for future research. It is, therefore, advisable for those wishing to undertake future studies on the subject to collect qualitative data that could help to identify attitudes and skills that would not otherwise emerge. This information would be useful to further differentiate the results obtained. In spite of this limitation, the survey contributes to increasing the current literature and evidence on the attitudes and skills of nurse middle managers.

**Implications: Education and Training**

Training programs focused on time management can equip nurses with the skills necessary to handle their demanding workloads. Incorporating time management training into nursing education can prepare future nurses to manage their responsibilities efficiently. A study by Glantz et al. [25] emphasized the importance of integrating time management skills into nursing curricula to enhance the preparedness of nursing graduates.

As analyzed by Michel et al. [26] nurses should aim at optimizing three main areas: (i) the greater involvement of RNs in building care relationships and the direct care of patients and relatives; (ii) better use of their skills in their autonomous nursing role, including making clinical nursing assessments and providing therapeutic education, and (iii) more systematically updating nurses’ knowledge. Not all nursing coordinators and middle managers manage to acquire and put into practice in every situation all these attitudes and skills they should have. A correct use of time management inevitably leads to better management of care.

In agreement with the study of Orzano et al. [27] knowledge management facilitates individual and organizational decision (time management) making or sensemaking and learning to achieve an organization’s mission and enhance organizational performance. Setting priorities is another tactic, which is a highly useful method of maximizing TM. Mirroring our results, Malkoc SA et al. [28] claimed that nurse management can better organize their calendar by concentrating on the activities that are more urgent.

## 6. Conclusions

Work-related stress has increased considerably in recent years, and this has inevitably also affected the lives of nurse managers. It is important for nurses to be more conscious of how they spend their time, recognize and deal with time wasters, and develop their time management abilities. Delegation, prioritization, planning, and many other techniques that can assist managers in striking a better balance between their work and home lives are undoubtedly the most popular time management (TM) practices.

Healthcare institutions have a duty to prioritize healthy work settings and to encourage team member health in addition to providing safe and hazard-free work environments. Therefore, a healthy workplace incorporates health promotion to enhance employees’ well-being. Well-being at the work level influences all the various aspects of the workers involved. Surely further studies that could relate the two issues would give a broader view of the phenomenon.

## Figures and Tables

**Table 1:** Sociodemographic characteristics of participants.

**Sex**	Female	67.6%
	Male	32.4%
**Employment**	Nurse clinician	75.7%
	Acting head nurse	8.1%
	Ward coordinator	12.2%
	Department coordinator	1.4%
	Other	2.7%
**Qualification**	High school diploma	21.6%
	Bachelor’s degree	54.1%
	Master’s degree	23%
	PhD	1.4%
**Type of work**	Full-time	97.3%
	Part-time	2.7%
**Type of hospital**	Private	21.6%
	Public	78.4%
**Children**	Yes	64.9%
	No	35.1%
**Age**	<30 years old	10.8%
	31–40 years old	21.6%
	41–50 years old	18.9%
	51–60 years old	40.5%
	61–70 years old	8.1%

**Table 2:** Time management.

Item	Completely Disagree	Indifferent	Completely Agreed
***I break down complex and difficult projects into smaller, more manageable tasks***	13.5%	25.7%	60.8%
***I set myself deadlines when I set out to accomplish a task***	9.5%	21.6%	68.9%
***I set priorities to determine the time order in which I will carry out different tasks***	2.7%	21.7%	75.6%
***I plan my activities at least one week in advance***	36.5%	37.9%	25.6%
***My working days are so unpredictable that I cannot plan and manage my time in the long term***	43.2%	39.2%	17.6%
***I feel in control of my time***	31.1%	40.5%	28.4%
***I have to spend a lot of my time on unimportant tasks***	55.4%	29.7%	14.9%

**Table 3:** Health condition.

Item	As Usual	Less than Usual	Much More than Usual	Not at All	More than Usual
***Concentration problems***	32.4%	17.6%	1.4%	29.7%	18.9%
***Loss of sleep***	32.4%	10.8%	5.4%	28.4%	23%
***Feeling to perform unhelpful activities***	20.3%	21.6%	4.1%	43.2%	10.8%
***Difficulty making decisions***	28.4%	17.6%	2.7%	43.2%	8.1%
***Feeling under stress***	33.8%	13.5%	14.9%	8.1%	29.7%
***Difficulty overcoming problems***	36.5%	20.3%	5.4%	21.6%	16.2%
***Lack of pleasure in performing many activities***	28.4%	18.9%	6.8%	31.1%	14.9%
***Difficulty in coping with problems***	37.8%	18.9%	4.1%	28.4%	10.8%
***Unhappy and depressed***	18.9%	13.5%	2.7%	58.1%	6.8%
***Loss of self-confidence***	20.3%	21.6%	2.7%	50%	5.4%

**Table 4:** Ability to delegate.

Item	Never	Sometimes	Always
***I delegate at the last second***	67.6%	23%	9.4%
***When a task is related to my priorities, I avoid delegating it***	16.2%	18.9%	64.9%
***I use delegation to develop employees’ competences***	32.4%	31.1%	36.5%

## Data Availability

The data that support the findings of this study are available from the corresponding author upon reasonable request. Due to privacy concerns, data are not publicly available but can be accessed by contacting corresponding author.

## Supplementary Materials

The following supporting information can be downloaded at: https://www.mdpi.com/article/10.3390/nursrep14030157/s1, Supplementary File: STROBE Statement—checklist of items that should be included in reports of observational studies.

## Public Involvement Statement

There was no public involvement in any aspect of this research.

## Guidelines and Standards Statement

This manuscript was drafted against the STROBE Strengthening the Reporting of Observational studies in Epidemiology) and CHERRIES (Checklist for Reporting Results of Internet E-Surveys) criteria.
